# Association between Computed Tomographic Biomarkers of Cerebral Small Vessel Diseases and Long‐Term Outcome after Spontaneous Intracerebral Hemorrhage

**DOI:** 10.1002/ana.25949

**Published:** 2020-11-20

**Authors:** Mark A. Rodrigues, Neshika E. Samarasekera, Christine Lerpiniere, Luke A. Perry, Tom J. Moullaali, James J.M. Loan, Joanna M. Wardlaw, Rustam Al‐Shahi Salman, for the Lothian Audit of the Treatment of Cerebral Haemorrhage Collaborators

**Affiliations:** ^1^ Centre for Clinical Brain Sciences University of Edinburgh Edinburgh UK; ^2^ Department of Neuroradiology NHS Lothian Edinburgh UK; ^3^ Department of Anaesthesia and Pain Management Royal Melbourne Hospital Melbourne Victoria Australia; ^4^ Department of Clinical Neurosciences NHS Lothian Edinburgh UK; ^5^ Centre for Discovery Brain Sciences University of Edinburgh Edinburgh UK; ^6^ UK Dementia Research Institute at the University of Edinburgh Edinburgh UK; ^7^ Row Fogo Centre for Research into Ageing and the Brain University of Edinburgh Edinburgh UK

## Abstract

**Objective:**

A study was undertaken to assess whether cerebral small vessel disease (SVD) computed tomographic (CT) biomarkers are associated with long‐term outcome after intracerebral hemorrhage.

**Methods:**

We performed a prospective, community‐based cohort study of adults diagnosed with spontaneous intracerebral hemorrhage between June 1, 2010 and May 31, 2013. A neuroradiologist rated the diagnostic brain CT for acute intracerebral hemorrhage features and SVD biomarkers. We used severity of white matter lucencies and cerebral atrophy, and the number of lacunes to calculate the CT SVD score. We assessed the association between CT SVD biomarkers and either death, or death or dependence (modified Rankin Scale scores = 4–6) 1 year after first‐ever intracerebral hemorrhage using logistic regression, adjusting for known predictors of outcome.

**Results:**

Within 1 year of intracerebral hemorrhage, 224 (56%) of 402 patients died. In separate models, 1‐year death was associated with severe atrophy (adjusted odds ratio [aOR] = 2.54, 95% confidence interval [CI] = 1.44–4.49, *p* = 0.001) but not lacunes or severe white matter lucencies, and CT SVD sum score ≥ 1 (aOR = 2.50, 95% CI = 1.40–4.45, *p* = 0.002). Two hundred seventy‐seven (73%) of 378 patients with modified Rankin Scale data were dead or dependent at 1 year. In separate models, 1‐year death or dependence was associated with severe atrophy (aOR = 3.67, 95% CI = 1.71–7.89, *p* = 0.001) and severe white matter lucencies (aOR = 2.18, 95% CI = 1.06–4.51, *p* = 0.035) but not lacunes, and CT SVD sum score ≥ 1 (aOR = 2.81, 95% CI = 1.45–5.46, *p* = 0.002).

**Interpretation:**

SVD biomarkers on the diagnostic brain CT are associated with 1‐year death and dependence after intracerebral hemorrhage, independent of known predictors of outcome. ANN NEUROL 2021;89:266–279

Spontaneous intracerebral hemorrhage (ICH) accounts for 9 to 27% of strokes worldwide and is the most severe type of stroke.^1^ One‐month and 1‐year case fatalities are around 40 and 55%, respectively.^1^, ^2^ Survivors of ICH attributed to cerebral small vessel diseases (SVDs) are often left disabled, with 16 to 46% functionally dependent on others at 6 months, and 43 to 46% are dependent at 1 year.^3^

Determining risk factors for poor outcome after ICH is important to inform discussions about prognosis with patients and their carers, and to guide treatment decisions. Well‐established risk factors for poor outcome after ICH include increasing age, decreasing conscious level on admission, and features of ICH, such as location, increasing volume, and the presence of intraventricular hemorrhage.^3^, ^4^ Most of these risk factors represent the severity and consequences of ICH, but outcome may also be determined by the brain in which ICH occurs.

We hypothesized that the severity of SVD on brain imaging may be a risk factor for death or dependence after ICH. About 85% of ICHs are attributed to SVDs, which usually underlie such ICHs.^5^, ^6^ SVDs may have many subsequent manifestations other than recurrent ICH, such as dementia^7^, ^8^ and ischemic stroke.^9^ Furthermore, SVD biomarkers are associated with physical disabilities in elderly patients without ICH^10^ and poor 90‐day and 6‐month functional outcome after ischemic stroke.^11^, ^12^

White matter lucencies (WMLs) on brain computed tomography (CT) were associated with poor 28‐ and 90‐day functional outcome and 90‐day death after SVD‐ICH in a recent meta‐analysis of 9 studies, which may have been affected by selection bias.^13^ Less is known about the association of not only WMLs but also other individual CT SVD biomarkers (such as lacunes and cerebral atrophy^14^, ^15^, ^16^) with longer‐term outcomes after SVD‐ICH. Composite SVD‐burden scores have shown added prognostic value over individual SVD biomarkers in other settings^11^ ^,^ ^17^, ^18^, ^19^, ^20^, ^21^; however, the association of the CT SVD score^22^ with SVD‐ICH outcome is unknown.

We aimed to assess the associations of SVD biomarkers on the first brain CT to diagnose spontaneous ICH—individually and as a composite CT SVD score—with 1‐year death or dependence after first‐ever SVD‐ICH in a prospective, community‐based cohort study.

## Patients and Methods

### *Study Design, Setting, and Patients*

We performed a community‐based inception cohort study of patients with spontaneous ICH living in the Lothian health board region of Scotland (Lothian Audit of the Treatment of Cerebral Hemorrhage [LATCH]).^5^ We prospectively identified all incident cases of spontaneous ICH in adults (aged ≥16 years) between June 1, 2010 and May 31, 2013 inclusive using multiple overlapping sources of case ascertainment.^5^ For this study, we excluded patients with ICH secondary to an underlying macrovascular or structural cause other than SVDs, patients with a previous symptomatic ICH, and patients without a diagnostic noncontrast brain CT.

The NHS Lothian Caldicott Guardian approved LATCH (reference number 1501). Patients in NHS Lothian were informed about the use of their data for audit, and information leaflets about LATCH were distributed to inform patients and their carers about their right to opt out. Analysis of an anonymized dataset did not require research ethics committee approval.

### *Risk Factors*

We collected demographics and the presence of relevant comorbidities, baseline clinical data, and medication use at the time of ICH by interviewing patients and their families at the time of presentation and reviewing primary and secondary care records.^5^

One neuroradiologist (M.A.R.) reformatted the diagnostic noncontrast brain CT into standard axial, coronal, and sagittal planes and used a standardized pro forma derived from large‐scale stroke studies to assess the images as previously described.^6^ M.A.R. evaluated the number and location of acute ICHs,^23^ the ICH volume using an ABC/2 approach,^24^ and the presence of extra‐axial hemorrhage (in subarachnoid, subdural, or intraventricular spaces). M.A.R. graded anterior and posterior WMLs separately as absent (0), lucency restricted to the periventricular white matter (1), or lucency covering the entire region from the lateral ventricle to the cortex (2) using the van Swieten scale.^25^ M.A.R. separately rated cortical and central atrophy as none (0), moderate (1), or severe (2) against a standard template.^11^ M.A.R. assessed WMLs and atrophy in the cerebral hemisphere contralateral to the ICH to reduce the effect of perihematomal edema and local mass effect. M.A.R. counted the number of lacunes, defined as a round or ovoid subcortical hypoattenuating lesion between 3 and 15mm in diameter in the territory of 1 perforating arteriole.^26^ MAR performed the CT reformatting and ratings using Carestream Vue PACS (v11.3.2; Carestream Health, Rochester, NY), masked to clinical and outcome information.

**FIGURE 1:**
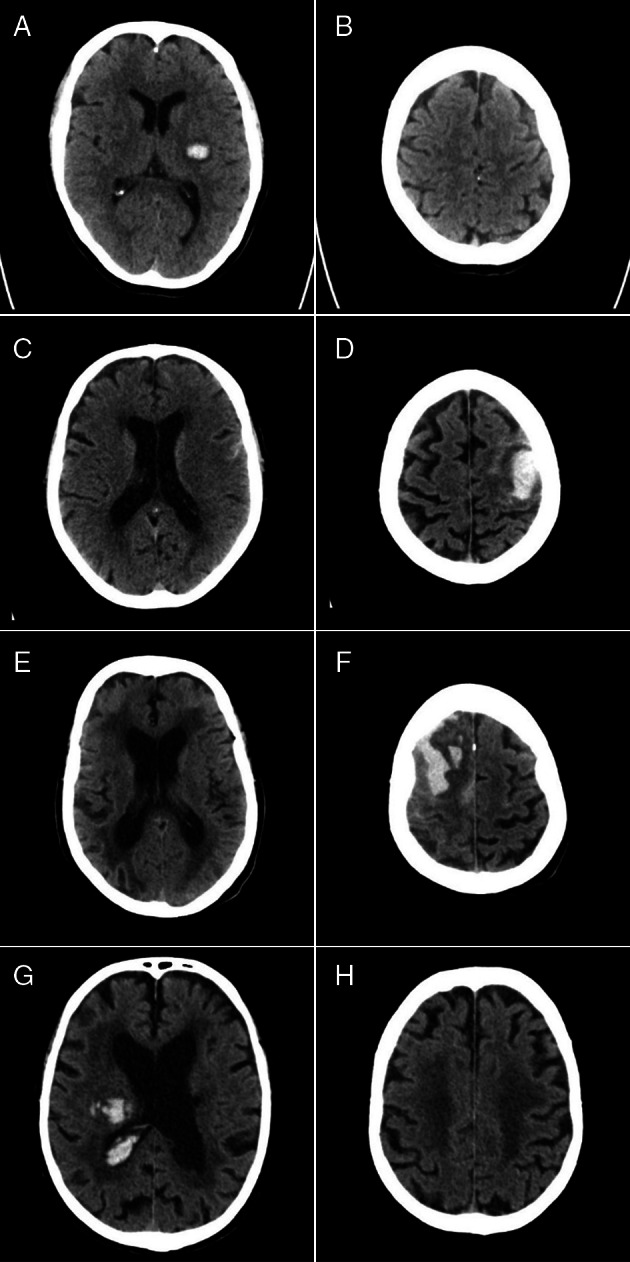
Example axial noncontrast brain computed tomographic (CT) images at the level of the lateral ventricular choroid plexus (A, C, E, and G) and centrum semiovale (B, D, F, and H) showing the range of CT small vessel disease (SVD) scores in patients with first‐ever SVD–intracerebral hemorrhage (ICH). (A, B) CT SVD score 0 (anterior white matter lucencies [WMLs] = 0, posterior WMLs = 0, no lacunes, central atrophy = 0, cortical atrophy = 0). (C, D) CT SVD 1 (anterior WMLs = 1, posterior WMLs = 1, no lacunes, central atrophy = 1, cortical atrophy = 2). (E, F) CT SVD 2 (anterior WMLs = 2, posterior WMLs = 2, no lacunes, central atrophy = 2, cortical atrophy = 2). (G, H) CT SVD 3 (anterior WMLs = 2, posterior WMLs = 2, 4 lacunes [one of which is visible in the left corona radiata], central atrophy = 2, cortical atrophy = 1).

We calculated the CT SVD sum score^22^ by awarding 1 point for each of the following: (1) severe (=2) WMLs in the anterior or posterior periventricular white matter, (2) ≥2 lacunes, and (3) severe (=2) central or cortical atrophy. The ordinal sum score quantifies the global burden of SVD (Fig 1) from 0 (no imaging features of severe SVD) to 3 (all 3 severe imaging features of SVD).

### *Outcomes*

We used multiple sources of information to follow up patients, including annual primary care practitioner postal questionnaires, yearly review of NHS Lothian's secondary care electronic records system, and evaluation of primary care records for all patients who died.^5^

We assessed outcome 1 year after ICH to allow functional recovery from the consequences of acute ICH during the first year afterward.^3^, ^27^ We prespecified 2 outcomes: (1) death from any cause; and (2) death or dependence, which we defined—as others have done, in view of the spectrum of severity of outcome after ICH^28^, ^29^, ^30^, ^31^—as a modified Rankin Scale score of 4 to 6.^32^

A consultant vascular neurologist (R.A.‐S.S.) confirmed the cause and date of death using the death certificate and primary care and hospital records, masked to the CT ratings. The 1‐year modified Rankin Scale score was ascertained in surviving patients with a postal questionnaire sent to the patients' primary care practitioners. The primary care practitioners used all available clinical information to derive the modified Rankin Scale score, unaware of this study of CT ratings of SVD.

### *Sample Size*

We used the largest sample size possible from a 3‐year community‐based cohort study to maximize power and generalizability. We reduced overfitting by prespecifying risk factors and building multivariable models with at least 10 outcome events per risk factor.^33^

### *Missing Data*

We excluded patients with missing risk factors or outcomes from the relevant analyses due to the infrequency of missing data.

### *Statistical Analysis*

For the 2 outcomes, death and death or dependence at 1 year after ICH, we compared the frequency of clinical characteristics and diagnostic noncontrast brain CT features between groups using the χ^2^ test for categorical variables and the Mann–Whitney *U* test for non‐normally distributed continuous variables. We corrected for multiple comparisons for each outcome using the Benjamini and Hochberg method.^34^

We performed multivariable logistic regression to assess whether CT SVD biomarkers were associated with each outcome independent of prespecified predictors. We included 10 prespecified predictors (age, sex, pre‐ICH diagnosis of dementia, pre‐ICH diagnosis of diabetes, antiplatelet and anticoagulant use at the time of the ICH, admission Glasgow Coma Scale [GCS] score, location of the largest ICH [lobar vs nonlobar (deep and infratentorial)], volume of the largest ICH, and intraventricular hemorrhage) based on the variables most frequently associated with long‐term survival and functional outcome after ICH.^3^, ^35^ In the first model (separate CT SVD biomarkers model), we also included the 3 components of the CT SVD score (severe anterior or posterior WMLs, severe cortical or central atrophy, and ≥2 lacunes) as separate covariates. In the second model (CT SVD sum score model), we included the composite CT SVD score alone, dichotomized (0 vs ≥1) to differentiate patients with (CT SVD score ≥ 1) and without (CT SVD score = 0) evidence of severe SVD on CT. We modeled the continuous predictors age, GCS score on admission, and ICH volume as linear associations, as there was no evidence of nonlinearity. We did not include any interaction terms, as there was no evidence of any interaction between ICH location, ICH volume, and CT SVD score.

We compared the multivariable logistic regression models against the ICH score^4^ for the 2 outcomes using the *c* statistic and DeLong's test for 2 correlated receiver operating characteristic curves.

The use of do not attempt resuscitation orders, withdrawal of active care, and the provision of rehabilitation, which are associated with death and functional outcome after ICH,^36^, ^37^, ^38^, ^39^ may have been influenced by some of the predictors included in the models. Therefore, we performed post hoc sensitivity analyses in patients who survived at least 30 days after their first‐ever ICH. Patients with a pre‐ICH modified Rankin Scale score of 4 or 5 are unlikely to improve their function after ICH. Therefore, we performed post hoc sensitivity analyses in patients with a pre‐ICH modified Rankin Scale score of 0 to 3.

We performed the statistical analyses using R statistical package version 3.4.4.

## Results

**FIGURE 2:**
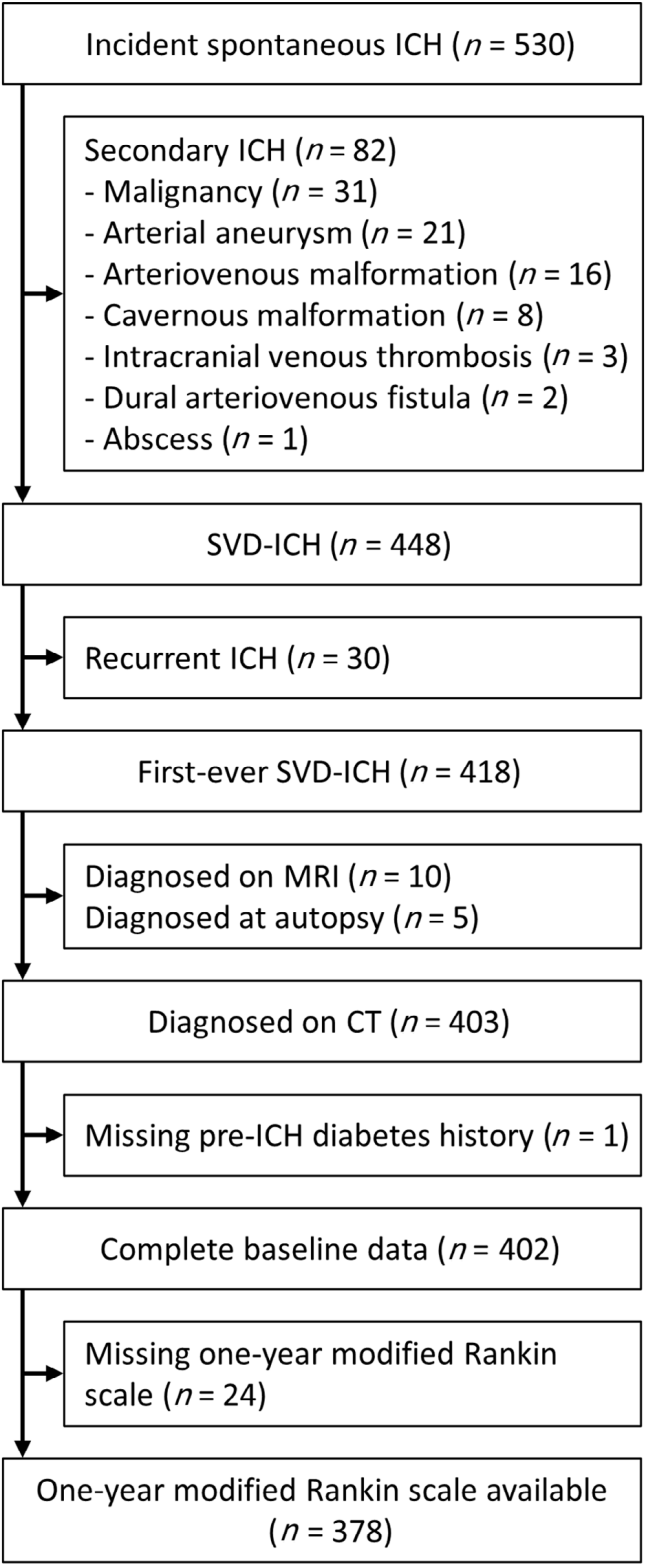
Flow of patients. CT = computed tomography; ICH = intracerebral hemorrhage; MRI = magnetic resonance imaging; SVD = small vessel disease.

Between June 1, 2010 and May 31, 2013, there were 530 patients with incident spontaneous ICH, 418 of whom had a first‐ever SVD‐ICH. We included 402 (96%) with baseline diagnostic noncontrast brain CT and complete baseline data (Fig 2). The median age was 78 years (interquartile range [IQR] = 68–84), and 185 were men (46%). The median time between ICH onset and CT was 0 days (IQR = 0–1). One hundred ninety‐nine had a lobar ICH, 152 a deep ICH, and 51 an infratentorial ICH. The median ICH volume was 20ml (IQR = 6–55), and 188 (47%) had intraventricular hemorrhage.

### *One‐Year Death after First‐Ever SVD‐ICH*

Two hundred twenty‐four patients (56%) were dead at 1 year after their first‐ever ICH, whereas 178 patients were alive. Patients who died were older, with a more frequent history of pre‐ICH ischemic stroke, and had higher pre‐ICH modified Rankin Scale scores and lower GCS scores on admission on univariate analysis (Table 1). Larger ICH volume and the presence of intraventricular hemorrhage and subarachnoid hemorrhage were also associated with death at 1 year, as were increasing severity of WMLs and atrophy and more severe CT SVD score (Table 2).

**TABLE 1:** Baseline Clinical Features in First‐Ever SVD‐ICH Patients Who Were Alive at 1 Year after First‐Ever ICH versus Those Who Were Dead

Feature	Alive at 1 Year, n = 178		Dead at 1 Year, n = 224		*p* ^a^
Age, yr	74	(61–82)	79	(72–84)	<0.001
Sex					
Female	100	(56)	117	(52)	0.530
Male	78	(44)	107	(48)	
Comorbidities					
Hypertension	112	(63)	153	(68)	0.371
Ischemic stroke	20	(11)	48	(21)	0.015
Transient ischemic attack	16	(9)	29	(13)	0.322
Dementia	16	(9)	36	(16)	0.063
Diabetes	16	(9)	30	(13)	0.269
Atrial fibrillation	39	(22)	50	(22)	0.951
Myocardial infarction	11	(6)	26	(12)	0.104
Hyperlipidemia	28	(16)	43	(19)	0.487
Smoking status^b^					
Current	37	(21)	51	(23)	0.488
Ex‐smoker	59	(33)	84	(38)	
Never	82	(46)	87	(39)	
Pre‐ICH modified Rankin Scale^c^					
0	81	(46)	48	(22)	
1	37	(21)	46	(21)	
2	30	(17)	58	(26)	
3	25	(14)	55	(25)	
4	5	(3)	10	(5)	
5	0	(0)	3	(1)	
Pre‐ICH modified Rankin Scale	2	(1–3)	3	(2–4)	<0.001
Medications on admission					
Antiplatelet drug(s)	72	(40)	103	(46)	0.371
Anticoagulant drug(s)	22	(12)	32	(14)	0.656
Antihypertensive drug(s)	86	(48)	111	(50)	0.859
Admission GCS score	14	(14–15)	11	(6–14)	<0.001

Data are n (%) or median (interquartile range).

^a^ Adjusted for multiple comparisons using the Benjamini and Hochberg method.

^b^ Data missing in 2 patients.

^c^ Data missing in 4 patients.

GCS = Glasgow coma scale; ICH = intracerebral hemorrhage; SVD = small vessel disease.

**TABLE 2:** Diagnostic Noncontrast Brain CT Features in First‐Ever SVD‐ICH Patients Who Were Alive at 1 Year after First‐Ever ICH versus Those Who Were Dead

Feature	Alive at 1 Year, n = 178		Dead at 1 Year, n = 224		*p* ^a^
ICH location					
Lobar	91	(51)	108	(48)	0.650
Deep	68	(38)	84	(38)	
Infratentorial	19	(11)	32	(14)	
ICH volume, ml	10	(3–22)	38	(13–82)	<0.001
Intraventricular hemorrhage	48	(27)	140	(63)	<0.001
Subarachnoid hemorrhage	65	(37)	112	(50)	0.015
Subdural hemorrhage	12	(7)	30	(13)	0.057
Fingerlike projections	11	(6)	33	(15)	0.015
Number of lacunes	0	(0–1)	0	(0–1)	0.726
≥2 lacunes	33	(19)	41	(18)	0.952
Anterior WMLs					
0	42	(24)	37	(17)	0.003
1	99	(56)	103	(46)	
2	37	(21)	84	(38)	
Posterior WMLs					
0	71	(40)	53	(24)	0.003
1	41	(23)	52	(23)	
2	66	(37)	119	(53)	
Severe (=2) anterior or posterior WMLs	69	(39)	128	(57)	0.001
Central atrophy					
0	56	(32)	45	(20)	<0.001
1	109	(61)	131	(59)	
2	13	(7)	48	(21)	
Cortical atrophy					
0	51	(29)	54	(24)	0.057
1	99	(56)	110	(49)	
2	28	(16)	60	(27)	
Severe (=2) central or cortical atrophy	37	(21)	91	(41)	<0.001
CT SVD score					
0	82	(46)	54	(24)	<0.001
1	60	(34)	94	(42)	
2	29	(16)	62	(28)	
3	7	(4)	14	(6)	
CT SVD score ≥ 1	96	(54)	170	(76)	<0.001

Data are n (%) or median (interquartile range). ICH location and volume relate to the largest ICH if there were multiple acute ICHs on the diagnostic brain CT.

^a^ Adjusted for multiple comparisons using the Benjamini and Hochberg method.

CT = computed tomography; ICH = intracerebral hemorrhage; SVD = small vessel disease; WML = white matter lucency.

In the prespecified multivariable separate CT SVD biomarkers model (Table 3), the presence of severe atrophy was independently associated with death at 1 year after adjusting for known predictors of long‐term survival in ICH (adjusted odds ratio [aOR] = 2.54, 95% confidence interval [CI] = 1.44–4.49, *p* = 0.001). Severe WMLs showed a borderline significant independent association (aOR = 1.75, 95% CI = 0.99–3.08, *p* = 0.053), whereas there was no significant association between the presence of at least 2 lacunes and death. In the prespecified multivariable CT SVD sum score model (see Table 3), CT SVD score ≥ 1 was independently associated with death at 1 year after adjusting for known predictors of long‐term survival in ICH (aOR = 2.50, 95% CI = 1.40–4.45, *p* = 0.002). Increasing age, male sex, decreasing admission GCS score, nonlobar ICH location, increasing ICH volume, and the presence of intraventricular hemorrhage were independently associated with death at 1 year in both models.

**TABLE 3:** Multivariable Models for Death at 1 Year after First‐Ever ICH in First‐Ever SVD‐ICH

	β Coefficient (standard error)		Odds Ratio (95% CI)		*p*
Separate CT SVD biomarkers model					
Intercept	−2.64	(1.21)			
Age, per year increase	0.04	(0.01)	1.05	(1.02–1.07)	<0.001
Male sex	0.70	(0.28)	2.02	(1.17–3.49)	0.011
Pre‐ICH dementia	−0.16	(0.41	0.85	(0.38–1.92)	0.703
Pre‐ICH diabetes	0.58	(0.42)	1.79	(0.78–4.10)	0.168
Antiplatelet use at ICH	−0.54	(0.31)	0.59	(0.32–1.07)	0.080
Anticoagulant use at ICH	−0.14	(0.42)	0.87	(0.38–1.97)	0.739
Admission GCS score, per point increase	−0.21	(0.05)	0.81	(0.74–0.89)	<0.001
Nonlobar ICH location	0.68	(0.30)	1.97	(1.10–3.55)	0.023
ICH volume, per ml increase	0.03	(0.01)	1.03	(1.02–1.04)	<0.001
Intraventricular hemorrhage	0.63	(0.29)	1.88	(1.06–3.31)	0.030
≥2 lacunes	−0.04	(0.34)	0.96	(0.50–1.86)	0.913
Severe anterior or posterior WMLs	0.56	(0.29)	1.75	(0.99–3.08)	0.053
Severe central or cortical atrophy	0.93	(0.29)	2.54	(1.44–4.49)	0.001
CT SVD sum score model					
Intercept	−3.07	(1.21)			
Age, per year increase	0.05	(0.01)	1.05	(1.03–1.08)	<0.001
Male sex	0.71	(0.27)	2.03	(1.19–3.47)	0.010
Pre‐ICH dementia	−0.13	(0.41)	0.87	(0.39–1.96)	0.746
Pre‐ICH diabetes	0.44	(0.42)	1.55	(0.69–3.49)	0.294
Antiplatelet use at ICH	−0.48	(0.30)	0.62	(0.35–1.12)	0.111
Anticoagulant use at ICH	−0.09	(0.42)	0.91	(0.40–2.08)	0.827
Admission GCS score, per point increase	−0.21	(0.05)	0.81	(0.73–0.89)	<0.001
Nonlobar ICH location	0.67	(0.30)	1.95	(1.09–3.47)	0.024
ICH volume, per ml increase	0.03	(0.01)	1.03	(1.02–1.04)	<0.001
Intraventricular hemorrhage	0.63	(0.29)	1.87	(1.06–3.29)	0.030
CT SVD score ≥ 1	0.92	(0.30)	2.50	(1.40–4.45)	0.002

ICH location and volume relate to the largest ICH if there were multiple acute ICHs on the diagnostic brain CT. CI = confidence interval; CT = computed tomography; GCS = Glasgow Coma Scale; ICH = intracerebral hemorrhage; SVD = small vessel disease; WML = white matter lucency.

The ICH score had a *c* statistic of 0.79 (95% CI = 0.75–0.83) for 1‐year death. The *c* statistic of both the separate CT SVD biomarkers (0.86, 95% CI = 0.83–0.90, *p* = 0.010) and the CT SVD sum (0.86, 95% CI = 0.82–0.89, *p* = 0.013) multivariable models was significantly higher than the ICH score.

In sensitivity analyses of 235 patients who survived at least 30 days after their first‐ever ICH, severe atrophy (aOR = 2.24, 95% CI = 1.08–4.65, *p* = 0.030), and CT SVD score ≥ 1 (aOR = 2.47, 95% CI = 1.16–5.29, *p* = 0.020) remained independently associated with death at 1 year in separate multivariable models.

### *One‐Year Death or Dependence (Modified Rankin Scale Scores 4–6) after First‐Ever SVD‐ICH*

Three hundred seventy‐eight patients (94%) had complete modified Rankin Scale data at 1 year (see Fig 2). Patients with missing modified Rankin Scale outcome data had higher admission GCS, smaller ICH volumes, and less frequent intraventricular hemorrhage compared to those with complete modified Rankin Scale outcome data. The baseline clinical characteristics and diagnostic brain CT features were otherwise similar between the 2 groups.

The median modified Rankin Scale score 1 year after the index ICH was 2 (IQR = 1–3). One hundred one patients (26.7%) were alive and independent (modified Rankin Scale score = 0–3), and 277 patients (73.3%) were dead or dependent (modified Rankin Scale score = 4–6). Patients who were dead or dependent were older, and more frequently had a history of pre‐ICH ischemic stroke or diabetes, worse pre‐ICH modified Rankin Scale scores, and lower GCS score on admission on univariate analysis (Table 4). Larger ICH volume and the presence of intraventricular hemorrhage and subarachnoid hemorrhage were also associated with death or dependence at 1 year, as were increasing severity of WMLs and atrophy and more severe CT SVD score (Table 5).

**TABLE 4:** Baseline Clinical Features in First‐Ever SVD‐ICH Patients Who Were Dead or Dependent (Modified Rankin Scale = 4–6) at 1 Year after First‐Ever ICH versus Those Who Were Not (Modified Rankin Scale = 0–3)

Feature	Modified Rankin Scale = 0–3, n = 101		Modified Rankin Scale = 4–6, n = 277		*p* ^a^
Age, yr	72	(60–81)	79	(71–84)	<0.001
Sex					
Female	55	(54)	150	(54)	0.989
Male	46	(46)	127	(46)	
Comorbidities					
Hypertension	60	(59)	189	(68)	0.184
Ischemic stroke	9	(9)	57	(21)	0.017
Transient ischemic attack	11	(11)	33	(12)	0.865
Dementia	7	(7)	43	(16)	0.052
Diabetes	5	(5)	38	(14)	0.035
Atrial fibrillation	21	(21)	63	(23)	0.831
Myocardial infarction	7	(7)	30	(11)	0.406
Hyperlipidemia	15	(15)	51	(18)	0.584
Smoking status^b^					
Current	23	(23)	59	(22)	0.883
Ex‐smoker	34	(34)	102	(37)	
Never	44	(44)	114	(42)	
Pre‐ICH modified Rankin Scale^c^					
0	53	(53)	64	(23)	
1	22	(22)	59	(22)	
2	15	(15)	70	(26)	
3	11	(11)	63	(23)	
4	0	(0)	14	(5)	
5	0	(0)	3	(1)	
Pre‐ICH modified Rankin Scale	1	(1–3)	3	(2–4)	<0.001
Medications on admission					
Antiplatelet drug(s)	40	(40)	127	(46)	0.406
Anticoagulant drug(s)	12	(12)	37	(13)	0.831
Antihypertensive drug(s)	49	(49)	140	(51)	0.831
Admission GCS score	15	(14–15)	12	(8–14)	<0.001

Data are n (%) or median (interquartile range).

^a^ Adjusted for multiple comparisons using the Benjamini and Hochberg method.

^b^ Data missing in 2 patients.

^c^ Data missing in 4 patients.

GCS = Glasgow Coma Scale; ICH = intracerebral hemorrhage; SVD = small vessel disease.

**TABLE 5:** Diagnostic Noncontrast Brain CT Features in First‐Ever SVD‐ICH Patients Who Were Dead or Dependent (Modified Rankin Scale = 4–6) at 1 Year after First‐Ever ICH versus Those Who Were Not (Modified Rankin Scale = 0–3)

Feature	Modified Rankin Scale = 0–3, n = 101		Modified Rankin Scale = 4–6, n = 277		*p* ^a^
ICH location					
Lobar	50	(49)	137	(49)	0.999
Deep	38	(38)	104	(38)	
Infratentorial	13	(13)	36	(13)	
ICH volume, ml	7	(3–16)	33	(12–77)	<0.001
Intraventricular hemorrhage	19	(19)	167	(60)	<0.001
Subarachnoid hemorrhage	35	(35)	133	(48)	0.039
Subdural hemorrhage	8	(8)	32	(12)	0.406
Fingerlike projections	4	(4)	39	(14)	0.015
Number of lacunes	0	(0–1)	0	(0–1)	0.808
≥2 lacunes	16	(16)	50	(18)	0.808
Anterior WMLs					
0	31	(31)	44	(16)	0.002
1	51	(51)	135	(49)	
2	19	(19)	98	(35)	
Posterior WMLs					
0	51	(51)	68	(25)	<0.001
1	17	(17)	67	(24)	
2	33	(33)	142	(51)	
Severe (=2) anterior or posterior WMLs	34	(34)	152	(55)	0.001
Central atrophy					
0	35	(35)	61	(22)	<0.001
1	63	(62)	162	(59)	
2	3	(3)	54	(19)	
Cortical atrophy					
0	26	(26)	70	(25)	0.017
1	64	(63)	137	(50)	
2	11	(11)	70	(25)	
Severe (=2) central or cortical atrophy	14	(14)	105	(38)	<0.001
CT SVD score					
0	54	(54)	75	(27)	<0.001
1	33	(33)	113	(41)	
2	11	(11)	73	(26)	
3	3	(3)	16	(6)	
CT SVD score ≥ 1	47	(47)	202	(73)	<0.001

Data are n (%) or median (interquartile range). ICH location and volume relate to the largest ICH if there were multiple acute ICHs on the diagnostic brain CT.

^a^ Adjusted for multiple comparisons using the Benjamini and Hochberg method. CT = computed tomography.

ICH = intracerebral hemorrhage; SVD = small vessel disease; WML = white matter lucency.

In the prespecified multivariable separate CT SVD biomarkers model, severe atrophy (aOR = 3.67, 95% CI = 1.71–7.89, *p* = 0.001) and severe WMLs (aOR = 2.18, 95% CI = 1.06–4.51, *p* = 0.035) were independently associated with death or dependence at 1 year after adjusting for known predictors of long‐term functional outcome after ICH (Table 6). There was no significant association between the presence of at least 2 lacunes and death or dependence. In the prespecified multivariable CT SVD sum score model (see Table 6), CT SVD score ≥ 1 was independently associated with death or dependence at 1 year after adjusting for known predictors of long‐term survival in ICH (aOR = 2.81, 95% CI = 1.45–5.46, *p* = 0.002). Increasing age, pre‐ICH history of diabetes, decreasing admission GCS score, increasing ICH volume, and the presence of intraventricular hemorrhage were independently associated with death or dependence at 1 year in both models.

**TABLE 6:** Multivariable Models for Death or Dependence (Modified Rankin Scale = 4–6) at 1 Year after First‐Ever ICH in First‐Ever SVD‐ICH

	β Coefficient (standard error)		Odds Ratio (95% CI)		*p*
Separate CT SVD biomarkers model					
Intercept	−2.55	(1.74)			
Age, per year increase	0.06	(0.02)	1.06	(1.02–1.09)	0.001
Male sex	0.42	(0.36)	1.53	(0.76–3.07)	0.233
Pre‐ICH dementia	−0.38	(0.57)	0.68	(0.22–2.08)	0.502
Pre‐ICH diabetes	1.69	(0.60)	5.44	(1.67–17.71)	0.005
Antiplatelet use at ICH	−0.78	(0.37)	0.46	(0.22–0.95)	0.036
Anticoagulant use at ICH	−0.39	(0.53)	0.68	(0.24–1.90)	0.460
Admission GCS, per point increase	−0.24	(0.08)	0.79	(0.68–0.92)	0.002
Nonlobar ICH location	0.64	(0.35)	1.90	(0.96–3.77)	0.066
ICH volume, per ml increase	0.05	(0.01)	1.05	(1.03–1.07)	<0.001
Intraventricular hemorrhage	1.14	(0.38)	3.12	(1.48–6.58)	0.003
≥2 lacunes	0.02	(0.44)	1.02	(0.43–2.38)	0.972
Severe anterior or posterior WMLs	0.78	(0.37)	2.18	(1.06–4.51)	0.035
Severe central or cortical atrophy	1.30	(0.39)	3.67	(1.71–7.89)	0.001
CT SVD sum score model					
Intercept	−2.94	(1.71)			
Age, per year increase	0.06	(0.02)	1.07	(1.03–1.10)	<0.001
Male sex	0.47	(0.34)	1.59	(0.82–3.10)	0.170
Pre‐ICH dementia	−0.17	(0.55)	0.84	(0.29–2.44)	0.749
Pre‐ICH diabetes	1.45	(0.59)	4.25	(1.33–13.59)	0.015
Antiplatelet use at ICH	−0.66	(0.36)	0.52	(0.26–1.04)	0.064
Anticoagulant use at ICH	−0.22	(0.52)	0.80	(0.29–2.21)	0.670
Admission GCS score, per point increase	−0.25	(0.08)	0.78	(0.67–0.90)	0.001
Nonlobar ICH location	0.62	(0.34)	1.86	(0.95–3.65)	0.069
ICH volume, per ml increase	0.05	(0.01)	1.05	(1.03–1.07)	<0.001
Intraventricular hemorrhage	1.11	(0.38)	3.02	(1.44–6.33)	0.003
CT SVD score ≥ 1	1.03	(0.34)	2.81	(1.45–5.46)	0.002

ICH location and volume relate to the largest ICH if there were multiple acute ICHs on the diagnostic brain CT. CI = confidence interval; CT = computed tomography; GCS = Glasgow Coma Scale; ICH = intracerebral hemorrhage; SVD = small vessel disease; WML = white matter lucency.

The ICH score had a *c* statistic of 0.82 (95% CI = 0.76–0.86) for 1‐year death or dependence. The *c* statistic of both the separate CT SVD biomarkers (0.91, 95% CI = 0.88–0.94, *p* = 0.001) and the CT SVD sum (0.90, 95% CI = 0.87–0.93, *p* = 0.002) multivariable models was significantly higher than the ICH score.

In a sensitivity analysis of 211 patients who survived at least 30 days after their first‐ever ICH, severe atrophy (aOR = 2.85, 95% CI = 1.26–6.48, *p* = 0.012) and CT SVD score ≥ 1 (aOR = 2.33, 95% CI = 1.16–4.68, *p* = 0.018) remained independently associated with 1‐year death or dependence. Severe WMLs approached but did not achieve a statistically significant association (aOR = 1.95, 95% CI = 0.90–4.20, *p* = 0.089).

Sensitivity analyses of 357 patients with a pre‐ICH modified Rankin Scale score of 0 to 3 showed similar significance, direction, and magnitude of the independent associations with 1‐year death or dependence, suggesting that pre‐ICH dependence on others did not affect the principal associations with outcome.

In post hoc sensitivity analyses trichotomizing ICH location (lobar [reference], deep or infratentorial) in the multivariable models, deep ICH location remained statistically significantly associated with 1‐year death and 1‐year death or dependence. Infratentorial location did not show a statistically significant association. However, the direction and magnitude of the nonsignificant association were similar to deep ICH.

## Discussion

This prospective, community‐based cohort study has shown that some CT biomarkers of SVD, both individually and as part of the CT SVD score, are independently associated with 1‐year death and death or dependence following first‐ever SVD‐ICH.

In line with previous studies, we found that increasing age and decreasing admission GCS score, as well as features of the acute ICH, such as increasing ICH volume and the presence of intraventricular hemorrhage, were independently associated with death and functional outcome at 1 year after SVD‐ICH.^3^ The associations of other variables—such as sex, pre‐ICH anticoagulant drug use, and ICH location—with long‐term outcome after ICH have varied between studies.^3^

Baseline SVD markers on both CT and magnetic resonance imaging (MRI) of the brain are associated with worse prognosis after stroke, individually and when combined as composite scores.^11^, ^12^, ^19^ The association between SVD imaging biomarkers and poor functional outcome was strongest in those presenting with lacunar stroke, a stroke subtype caused by SVD.^19^

We found that severe WMLs were associated with 1‐year death or dependence after adjusting for other known predictors of long‐term outcome after ICH. Severe WMLs approached but did not achieve a statistically significant association with death, which was probably due to a lack of power. These findings concur with previous data showing an association between WMLs and short and medium‐term outcome after ICH. In a recent meta‐analysis of 9 studies and 4,948 participants with ICH, WMLs on CT or MRI of the brain were associated with both death (OR = 1.59, 95% CI = 1.21–2.08) and worse functional outcome (OR = 1.40, 95% CI = 1.17–1.68) during short and medium‐term follow‐up (28–90 days).^13^ However, there was significant heterogeneity between the included studies, with several potential confounders, such as study design and mean ICH volume.

Two studies have shown an association between cerebral atrophy and 90‐day death or major dependence following ICH.^14^, ^15^ However, both had selection biases, because participants with severe ICH or pre‐ICH dependence were excluded. We show similar findings, with independent associations between severe atrophy and both 1‐year death and death or dependence. In contrast, cerebral atrophy was associated with a good 90‐day functional outcome in one small study.^16^ This conflicting result probably relates to differences in participant selection, as this study included a very specific subgroup of participants with moderate volume basal ganglia ICH.^16^ In our study, we included all patients with ICH regardless of pre‐ICH dependence, ICH location, and ICH severity.

Few studies have assessed the prognostic value of lacunes after ICH. One study did not identify a statistically significant association between lacunes and outcome after ICH.^14^ Similarly, we did not find an association between lacunes and 1‐year death and death or dependence.

In separate models, we showed that the composite CT SVD score was associated with both death and death or dependence at 1 year. Composite SVD burden scores have shown prognostic value over individual SVD biomarkers in ischemic stroke.^11^, ^17^, ^18^ Composite MRI SVD scores were associated with poor functional outcome at discharge^21^ and 90 days^20^ after ICH in 2 recent studies. However, both studies were small and had selection biases, and the analyses were at risk of overfitting. To the best of our knowledge, this is the first study showing an association between the CT SVD score and longer‐term clinically relevant outcomes after ICH.

The mechanisms underlying the association of WMLs and atrophy with outcome after ICH remain unclear. The severity of these biomarkers may indicate the frailty of the underlying brain, which could influence susceptibility to and recovery from ICH. Potential mechanisms include reduced connectivity and neural plasticity in WMLs, which can influence recovery.^40^, ^41^ WMLs and atrophy are associated with poststroke cognitive impairment in the elderly,^7^, ^8^, ^42^, ^43^ which in turn is a predictor for outcome after stroke.^44^

This study has strengths. We used data from a contemporary, prospective, community‐based ICH cohort, which used multiple overlapping sources of case ascertainment and had low levels of missing data, to reduce selection bias and increase generalizability. We restricted our analyses to patients with first‐ever SVD‐ICH to provide a standard inception point. We minimized information bias for CT risk factor assessment by standardizing imaging format and rating of the diagnostic noncontrast brain CT using a standardized pro forma masked to clinical and outcome data. Patients were followed up using multiple sources of data. To minimize overfitting, we prespecified the risk factors for the logistic regression models based on evidence from the literature,^3^, ^4^ prespecified the outcomes, and restricted the number of risk factors to ensure there were at least 10 outcomes per variable in all models. We performed sensitivity analyses, the results of which did not change our conclusions.

Our study has limitations. The 1‐year modified Rankin Scale score was rated using a postal questionnaire sent to the patients' primary care practitioners. The primary care practitioners may not have specifically assessed the patient for this purpose, which could influence the accuracy of these data.^45^ However, mental incapacity is common among survivors of ICH; therefore, the modified Rankin Scale may be difficult to obtain from the patient directly, and using carers to assess functional abilities for some patients could result in bias.^46^ Also, patients' perception of disability can vary according to their demographics, resulting in discrepancies in modified Rankin Scale scores compared with assessment by trained professionals.^47^ Mass effect from the acute ICH, acute hydrocephalus, and perihematomal edema on CT may impair the assessment of SVD biomarkers. We tried to minimize this effect by assessing WMLs and atrophy in the cerebral hemisphere contralateral to the acute ICH (see Fig 1). MRI has better accuracy than CT for demonstrating SVD biomarkers,^26^ and would provide a more comprehensive imaging SVD assessment. However, CT is the most frequent type of brain imaging to diagnose ICH and has few contraindications, unlike MRI. Our approach reflects clinical practice in many parts of the world and makes the results more generalizable. Finally, do not attempt resuscitation orders and withdrawal of active care are known predictors of death in those with ICH considered to have a poor prognosis ICH,^36^, ^37^, ^38^ whereas physical therapy and rehabilitation may improve functional outcome after ICH.^39^ Although there were no local guidelines for these interventions during the study period, their use may have been influenced by some of the variables included in the models, such as age, admission GCS score, and brain CT features.^36^, ^37^, ^38^ Therefore, although the measured outcomes may result from direct effects of the risk factors, we cannot exclude the differential effects of do not attempt resuscitation orders, withdrawal of active care, and the provision of rehabilitation according to CT SVD biomarkers.^36^, ^48^, ^49^ However, severe atrophy and CT SVD score ≥ 1 remained independently associated with 1‐year death and functional outcomes in sensitivity analyses restricted to those who survived at least 30 days.

In conclusion, SVD biomarkers on the diagnostic brain CT are associated with 1‐year death and dependence after ICH, independent of age, features of the acute ICH, and other known prognostic factors. Future studies should assess whether the associations of CT SVD biomarkers with outcome after ICH can be reproduced in larger cohorts and participants of different ethnicities.

## Author Contributions

M.A.R., N.S., J.M.W., and R.A.‐S.S. contributed to the conception and design of the study; M.A.R., N.S., C.L., L.A.P., T.J.M., and J.J.M.L. contributed to the acquisition and analysis of data; all authors contributed to drafting the text and preparing the figures.

## Potential Conflicts of Interest

Nothing to report.
