# Supplementary material for: Association between Computed Tomographic Biomarkers of Cerebral Small Vessel Diseases and Long‐Term Outcome after Spontaneous Intracerebral Hemorrhage
Source: Ann Neurol. 2020 Nov 20;89(2):266–79. doi: 10.1002/ana.25949 (PMC7894327; doi:10.1002/ana.25949)
Supplement: Supplementary file 1 [file ANA-89-266-s001.doc]

Lothian Audit of the Treatment of Cerebral Haemorrhage Collaborators

| **Participant** | **Affiliated Institution** |
| --- | --- |
| Anne Addison | Western General Hospital, Edinburgh |
| Kate Ahmad | Western General Hospital, Edinburgh |
| Syed Alhadad | Western General Hospital, Edinburgh |
| Peter Andrews | Western General Hospital, Edinburgh |
| Elaine Bisset | Western General Hospital, Edinburgh |
| Peter Bodkin | Western General Hospital, Edinburgh |
| Ralph Bouhaidar | Western General Hospital, Edinburgh |
| Paul Brennan | Western General Hospital, Edinburgh |
| Brian Campbell | Western General Hospital, Edinburgh |
| Siddharthan Chandran | Western General Hospital, Edinburgh |
| Helen Cook | Western General Hospital, Edinburgh |
| Richard Davenport | Western General Hospital, Edinburgh |
| Martin Dennis | Western General Hospital, Edinburgh |
| Chris Derry | Western General Hospital, Edinburgh |
| Katrina Dodds | Western General Hospital, Edinburgh |
| Fergus Doubal | Western General Hospital, Edinburgh |
| Susan Duncan | Western General Hospital, Edinburgh |
| Andrew Elder | Western General Hospital, Edinburgh |
| Mike Fitzpatrick | Western General Hospital, Edinburgh |
| Peter Foley | Western General Hospital, Edinburgh |
| Ioannis Fouyas | Western General Hospital, Edinburgh |
| Sudipto Ghosh | Western General Hospital, Edinburgh |
| Rod Gibson | Western General Hospital, Edinburgh |
| Claire Gordon | Western General Hospital, Edinburgh |
| Robin Grant | Western General Hospital, Edinburgh |
| Russell Hewett | Western General Hospital, Edinburgh |
| Fiona Hughes | Western General Hospital, Edinburgh |
| Mark Hughes | Western General Hospital, Edinburgh |
| David Hunt | Western General Hospital, Edinburgh |
| Neil Hunter | Western General Hospital, Edinburgh |
| James Ironside | Western General Hospital, Edinburgh |
| Imran Liaquat | Western General Hospital, Edinburgh |
| Colin Josephson | Western General Hospital, Edinburgh |
| Anant Kamat | Western General Hospital, Edinburgh |
| Susan Kealey | Western General Hospital, Edinburgh |
| Sarah Keir | Western General Hospital, Edinburgh |
| Gillian Kerr | Western General Hospital, Edinburgh |
| Simon Kerrigan | Western General Hospital, Edinburgh |
| Peter Keston | Western General Hospital, Edinburgh |
| Matthew King | Western General Hospital, Edinburgh |
| Richard Knight | Western General Hospital, Edinburgh |
| Elizabeth Macdonald | Western General Hospital, Edinburgh |
| Graham Mackay | Western General Hospital, Edinburgh |
| Donald Macleod | Western General Hospital, Edinburgh |
| Malcolm Macleod | Western General Hospital, Edinburgh |
| Conor Maguire | Western General Hospital, Edinburgh |
| Steven Makin | Western General Hospital, Edinburgh |
| Ashok Mathews | Western General Hospital, Edinburgh |
| Fiona Maxwell | Western General Hospital, Edinburgh |
| Stuart McClellan | Western General Hospital, Edinburgh |
| Tracey Millar | Western General Hospital, Edinburgh |
| Zoe Morris | Western General Hospital, Edinburgh |
| Tim Morse | Western General Hospital, Edinburgh |
| Colin Mumford | Western General Hospital, Edinburgh |
| Katherine Murray | Western General Hospital, Edinburgh |
| Lynn Myles | Western General Hospital, Edinburgh |
| Graham Nimmo | Western General Hospital, Edinburgh |
| Yi Ng | Western General Hospital, Edinburgh |
| Suvankar Pal | Western General Hospital, Edinburgh |
| Kristiina Rannikmae | Western General Hospital, Edinburgh |
| Jonathan Rhodes | Western General Hospital, Edinburgh |
| Jerard Ross | Western General Hospital, Edinburgh |
| Tim Russell | Western General Hospital, Edinburgh |
| Peter Sandercock | Western General Hospital, Edinburgh |
| Robin Sellar | Western General Hospital, Edinburgh |
| Mano Shanmuganathan | Western General Hospital, Edinburgh |
| Himanshu Shekhar | Western General Hospital, Edinburgh |
| Henry Simms | Western General Hospital, Edinburgh |
| Mara Sittampalam | Western General Hospital, Edinburgh |
| Colin Smith | Western General Hospital, Edinburgh |
| Hamza Soleiman | Western General Hospital, Edinburgh |
| Helen Spiers | Western General Hospital, Edinburgh |
| Patrick Statham | Western General Hospital, Edinburgh |
| Neo Stavrinos | Western General Hospital, Edinburgh |
| Jon Stone | Western General Hospital, Edinburgh |
| Joyce Stuart | Western General Hospital, Edinburgh |
| Cathie Sudlow | Western General Hospital, Edinburgh |
| David Summers | Western General Hospital, Edinburgh |
| Pat Taylor | Western General Hospital, Edinburgh |
| Antonia Torgersen | Western General Hospital, Edinburgh |
| Margarethe van Dijke | Western General Hospital, Edinburgh |
| Robert Walker | Western General Hospital, Edinburgh |
| Belinda Weller | Western General Hospital, Edinburgh |
| William Whiteley | Western General Hospital, Edinburgh |
| Ian Whittle | Western General Hospital, Edinburgh |
| Robert Will | Western General Hospital, Edinburgh |
| Wendy Young | Western General Hospital, Edinburgh |
| Judith Anderson | Royal Infirmary of Edinburgh |
| Seona Broadbent | Royal Infirmary of Edinburgh |
| Laura Butler | Royal Infirmary of Edinburgh |
| Dave Caesar | Royal Infirmary of Edinburgh |
| Patricia Cantley | Royal Infirmary of Edinburgh |
| Jonathan Carter | Royal Infirmary of Edinburgh |
| Gareth Clegg | Royal Infirmary of Edinburgh |
| Andrew Coull | Royal Infirmary of Edinburgh |
| Alastair Crosswaite | Royal Infirmary of Edinburgh |
| James Dear | Royal Infirmary of Edinburgh |
| Simon Dummer | Royal Infirmary of Edinburgh |
| Fiona Duncan | Royal Infirmary of Edinburgh |
| Trish Elder-Gracie | Royal Infirmary of Edinburgh |
| Kate Enright | Royal Infirmary of Edinburgh |
| Tom Fitzgerald | Royal Infirmary of Edinburgh |
| Jane Fothergill | Royal Infirmary of Edinburgh |
| Brian Frier | Royal Infirmary of Edinburgh |
| David Grant | Royal Infirmary of Edinburgh |
| Alasdair Gray | Royal Infirmary of Edinburgh |
| Simon Hart | Royal Infirmary of Edinburgh |
| Robin Henderson | Royal Infirmary of Edinburgh |
| Alan Jaap | Royal Infirmary of Edinburgh |
| Simon Leigh-Smith | Royal Infirmary of Edinburgh |
| Michael Jones | Royal Infirmary of Edinburgh |
| Moyra Masson | Royal Infirmary of Edinburgh |
| Lynn McCallum | Royal Infirmary of Edinburgh |
| Martin McKechnie | Royal Infirmary of Edinburgh |
| Graham McKillop | Royal Infirmary of Edinburgh |
| Gillian Mead | Royal Infirmary of Edinburgh |
| Wendy Morley | Royal Infirmary of Edinburgh |
| Billie Morrow | Royal Infirmary of Edinburgh |
| Frank Morrow | Royal Infirmary of Edinburgh |
| Jon Murchison | Royal Infirmary of Edinburgh |
| Ross Murphy | Royal Infirmary of Edinburgh |
| Jasmine Ng | Royal Infirmary of Edinburgh |
| Olayinka Ogundipe | Royal Infirmary of Edinburgh |
| Dilip Patel | Royal Infirmary of Edinburgh |
| Alison Pollock | Royal Infirmary of Edinburgh |
| Matthew Reed | Royal Infirmary of Edinburgh |
| Geraint Roberts | Royal Infirmary of Edinburgh |
| Johann Selvarajah | Royal Infirmary of Edinburgh |
| Randy Smith | Royal Infirmary of Edinburgh |
| Claire Stirling | Royal Infirmary of Edinburgh |
| Neil Turner | Royal Infirmary of Edinburgh |
| Matthew Wilson | Royal Infirmary of Edinburgh |
| Stanko Yordanov | Royal Infirmary of Edinburgh |
| Nicola Bell | St John’s Hospital, West Lothian |
| Sarah Chambers | St John’s Hospital, West Lothian |
| Sandra Dewar | St John’s Hospital, West Lothian |
| Donald Farquhar | St John’s Hospital, West Lothian |
| Ali Harmouche | St John’s Hospital, West Lothian |
| Ashok Jacob | St John’s Hospital, West Lothian |
| Katherine Jackson | St John’s Hospital, West Lothian |
| Anne Knox | St John’s Hospital, West Lothian |
| Jon McCafferty | St John’s Hospital, West Lothian |
| Sam Moultrie | St John’s Hospital, West Lothian |
| Latana Munang | St John’s Hospital, West Lothian |
| Donald Noble | St John’s Hospital, West Lothian |
| Scott Ramsay | St John’s Hospital, West Lothian |
| Linda Spence | St John’s Hospital, West Lothian |
| Bethany Threlfall | St John’s Hospital, West Lothian |
| Adrian Williams | St John’s Hospital, West Lothian |
| James Wilson | St John’s Hospital, West Lothian |
| Alastair Fitzgerald | Astley Ainslie and Liberton Hospitals, Edinburgh |
| Andrew Jamieson | Astley Ainslie and Liberton Hospitals, Edinburgh |
| Peter Lange | Astley Ainslie and Liberton Hospitals, Edinburgh |
| Andrew McIntosh | Astley Ainslie and Liberton Hospitals, Edinburgh |
| Lewis Morrison | Astley Ainslie and Liberton Hospitals, Edinburgh |
| Iain Todd | Astley Ainslie and Liberton Hospitals, Edinburgh |
